# Management of suspected primary *Toxoplasma gondii* infection in pregnant women in Norway: twenty years of experience of amniocentesis in a low-prevalence population

**DOI:** 10.1186/s12884-017-1300-1

**Published:** 2017-04-26

**Authors:** Gry Findal, Anne Helbig, Guttorm Haugen, Pål A. Jenum, Babill Stray-Pedersen

**Affiliations:** 1 University of Oslo, Institute of Clinical Medicine, Oslo, Norway; 2 Division of Gynaecology and Obstetrics, Oslo University Hospital, Oslo, Norway; 3 Department of Laboratory Medicine, Section of Medical Microbiology, Vestre Viken Hospital Trust, Drammen, Norway

**Keywords:** *Toxoplasma gondii*, Toxoplasma infection, Pregnancy, Antenatal, Amniocentesis, Congenital, Prenatal diagnosis, PCR, Toxoplasma antibodies

## Abstract

**Background:**

Primary infection with *Toxoplasma gondii* during pregnancy may pose a threat to the fetus. Women infected prior to conception are unlikely to transmit the parasite to the fetus. If maternal serology indicates a possible primary infection, amniocentesis for toxoplasma PCR analysis is performed and antiparasitic treatment given. However, discriminating between primary and latent infection is challenging and unnecessary amniocenteses may occur. Procedure-related fetal loss after amniocentesis is of concern. The aim of the present study was to determine whether amniocentesis is performed on the correct patients and whether the procedure is safe for this indication.

**Methods:**

Retrospective study analysing data from all singleton pregnancies (*n* = 346) at Oslo University Hospital undergoing amniocentesis due to suspected maternal primary toxoplasma infection during 1993–2013. Maternal, neonatal and infant data were obtained from clinical hospital records, laboratory records and pregnancy charts. All serum samples were analysed at the Norwegian Institute of Public Health or at the Toxoplasma Reference Laboratory at Oslo University Hospital. The amniocenteses were performed at Oslo University Hospital by experienced personnel. Time of maternal infection was evaluated retrospectively based on serology results.

**Results:**

50% (173) of the women were infected before pregnancy, 23% (80) possibly in pregnancy and 27% (93) were certainly infected during pregnancy. Forty-nine (14%) women seroconverted, 42 (12%) had IgG antibody increase and 255 (74%) women had IgM positivity and low IgG avidity/high dye test titre. Fifteen offspring were infected with toxoplasma, one of them with negative PCR in the amniotic fluid. Median gestational age at amniocentesis was 16.7 gestational weeks (GWs) (Q_1_ = 15, Q_3_ = 22), with median sample volume 4 ml (Q_1_ = 3, Q_3_ = 7). Two miscarriages occurred 4 weeks after the procedure, both performed in GW 13. One of these had severe fetal toxoplasma infection.

**Conclusions:**

Half of our study population were infected before pregnancy. In order to reduce the unnecessary amniocenteses we advise confirmatory serology 3 weeks after a suspect result and suggest that the serology is interpreted by dedicated multidisciplinary staff. Amniocentesis is safe and useful as a diagnostic procedure in diagnosing congenital toxoplasma infection when performed after 15 GW.

## Background

Infection caused by the parasite *Toxoplasma gondii* is usually asymptomatic in immune-competent humans, but primary infection during pregnancy may pose a threat to the fetus. The risk and severity of a fetal infection depend on the gestational age (GA) at the time of infection [1–3]. A fetal infection in the first trimester, which is rare due to the low mother-to-child transmission rate (<10%), often leads to serious clinical manifestations such as fetal death, hydrocephaly and intracerebral calcifications [3, 4]. The transmission rate is higher in the third trimester (50–70%), but the fetuses are affected less severely and are likely to have a subclinical infection or be asymptomatic at birth [1, 5, 6]. Nevertheless, up to one-third of infected children will develop complications—most often ocular lesions (chorioretinitis)—during the first years of life [3, 7]. It is therefore important to detect primary *T. gondii* infection during pregnancy.

The immune response elicited by a primary infection results in the parasite rapidly forming semi-dormant intracellular pseudocysts, inflicting a latent stage [4].

Primary maternal infection is suspected when positivity for immunoglobulin M (IgM) antibodies and low immunoglobulin G (IgG) avidity are present [8]. Latent maternal *T. gondii* infection can be detected indirectly through the demonstration of toxoplasma-specific IgG antibodies. However, discriminating between latent and primary infection is a challenge unless seroconversion is observed, since toxoplasma IgM positivity and low IgG avidity may persist for months or even years after primary infection [5, 9–12]. When a primary toxoplasma infection is suspected during pregnancy, the pregnant woman should start antiparasitic treatment (spiramycin or azithromycin). Further, amniocentesis for toxoplasma polymerase chain reaction (PCR) analysis of the amniotic fluid is recommended to confirm fetal infection and assess the need for prolonged treatment and follow-up [4]. A positive toxoplasma PCR will lead to the treatment being changed to pyrimethamine-sulphadiazine with folic acid, which is continued until birth [13, 14]. Procedure-related complications after amniocentesis—the most serious of which is fetal loss—are of concern [15–17]. The risk might be lower than for genetic indications, due to the smaller volume of fluid obtained, and the higher average GA at which the procedure is performed.

No systematic serological screening is performed in Norway due to the low prevalence of *T. gondii* in the pregnant population. Few studies have addressed the impact of diagnostic amniocentesis in similar settings. We do not know the complication rate in this context, and have never evaluated our diagnostic guidelines.

The present study therefore evaluated the use of amniocentesis in our setting with the aim of determining whether the procedure is performed on the correct patients and if it is safe for this indication. To address these issues we performed a retrospective study analysing data from all women with singleton pregnancy at Oslo University Hospital undergoing amniocentesis due to suspected maternal primary toxoplasma infection.

## Methods

### Study population

This retrospective study included all singleton pregnancies in which amniocentesis was performed at Oslo University Hospital as part of the diagnostic workup for serologically suspected primary *T. gondii* infection (*n* = 346). The inclusion period was from 1 September 1992 to 31 December 2013. The included women were mainly referred from primary health-care centres in the South-East Health Region, which constitutes about 50% of the pregnant population in Norway [18]. The patients had been serologically tested because of risk factors, symptoms or (more commonly) at the initiative of the woman or her health-care provider.

Maternal, neonatal and infant data were obtained from clinical hospital records, laboratory records and pregnancy charts. Additional information was collected using a questionnaire sent to 40 women whose information on the date of delivery and birthweight was incomplete.

The GA was based on an ultrasound assessment performed early in second trimester or, if not available, on the first day of the last menstrual period. Maternal toxoplasma serology was mapped as the first and second serological tests performed during pregnancy, and a third serology was performed at birth. The second serological test was usually performed 3 weeks after the first to confirm the test result and to detect immunological changes [19]. The time of seroconversion, unknown in most patients, was set as the GA at the first positive toxoplasma antibody sample.

All amniocenteses were performed under ultrasound guidance; from 1992 to 1996 by experienced obstetricians, after 1996 by trained sub-specialists at the Section of Fetal Medicine at Oslo University Hospital.

### Laboratory methods

All samples were analysed at the Norwegian Institute of Public Health (before 2002) or at the Toxoplasma Reference Laboratory at Oslo University Hospital (established in 2002). The serum samples were analysed by indirect enzyme immunoassay (EIA) (Platelia Toxo IgG and Toxo IgM, Diagnostic Pasteur/Bio-Rad, Marnes-la-Coquette, France), microparticle enzyme immunoassay (MEIA) (Axsym, Abbott, Wiesbaden, Germany) or chemiluminescent microparticle immunoassay (CMIA) (Architect, Abbott, Wiesbaden, Germany) together with direct agglutination (DA IgG) and immunosorbent agglutination assays (ISAGA IgM and IgA) (Toxo-Screen DA and Toxo ISAGA, bioMérieux, Marcy l´Etoile, France) and dye test [20]. An antibody increase was defined as significant if there was at least a doubling in the IgG level expressed as IU/ml or as two-step titre increase.

Prior to 2005 the avidity method was performed as described previously using an in-house method based on the Platelia Toxo IgG assay [9], while a commercially available avidity test (Platelia Toxo IgG avidity, Bio-Rad) was used from 2005. The results obtained prior to 2005 were expressed as the percentage of antibodies resistant to elution by urea and from 2005 as an avidity index, with both of these measures being interpreted as low, borderline or high, according to a previous publication [9] or the manufacturer’s recommendation respectively.

In 1992 the B1-PCR method (PCR detecting the B1 gene of *T.gondii*) was introduced as a diagnostic indicator of antenatal toxoplasma infection [21]. Mouse inoculation was performed until 2002 as previously described [8, 21].

The presence of maternal toxoplasma infection was confirmed by either seroconversion of the toxoplasma IgG during pregnancy, or by a significant increase in IgG between the first and second toxoplasma samples [8]. The women were divided into three groups according to their toxoplasma serological profile: (i) IgG seroconversion, (ii) IgG increase or (iii) IgM positivity and low IgG avidity (dye test titre of >300 IU/ml was used before the IgG avidity test was established in 1996). In addition, the women were categorized retrospectively into three groups according to the suspected time of infection based on maternal and neonatal serology [8]: (i) infected before pregnancy, (ii) possibly infected during pregnancy and (iii) certainly infected during pregnancy. The categorisation was performed independently by two experienced investigators: a professor in microbiology (P.A.J.) and a professor in obstetrics (B.S.P.), both with expertise in toxoplasma diagnostics and infections during pregnancy. Consensus was reached for interpretation of the results.

The presence of congenital infection was confirmed by at least one of the following criteria being fulfilled: (i) positive toxoplasma PCR in amniotic fluid or neonatal cord blood, (ii) positive mouse inoculation of amniotic fluid or cord blood, (iii) positivity for toxoplasma IgM or immunoglobulin A (IgA) in postnatal serum, or (iv) toxoplasma IgG persisting in the infant at 12 months after birth [8].

### Statistical analysis

Group comparisons were done using different bivariate analyses depending on the type of variable and whether the variables conformed to a normal distribution. For all tests, a *p* value <0.05 was considered to indicate a statistically significant difference.

For GA at birth, miscarriages and induced abortions before 22 GWs were excluded.

Due to variation in missing information in the different variables, the denominators in the text may diverge from 346.

A database was constructed in EpiInfo (version 3.5.4, CDA, Atlanta, GA, USA) and the data were analysed and figures created in IBM SPSS Statistics (version 20.01, IBM Corporation, New York, NY, USA).

## Results

Our study included 346 women with singleton pregnancies whose characteristics are given in Table 1.

**Table 1:** Characteristics of 346 women in South-East Norway undergoing amniocentesis for suspected primary *Toxoplasma gondii* infection

	All cases *n* = 346		Infected before pregnancy *n* = 173		Possibly infected during pregnancy *n* = 80		Certainly infected during pregnancy *n* = 93	
Maternal age in years; mean (SD)	29.6 (5.3)		29.8 (5.3)		29.02 (5.1)		29.6 (5.4)	
Parity (*n* & %)								
P0	166	49.7	82	48.8	42	52.5	42	48.8
P ≥ 1	168	50.3	86	51.2	38	47.5	44	51.2
Missing information	12		5				7	
Nationality (*n* & %)								
Norwegian	281	81.2	143	82.7	63	78.8	75	80.6
Northern Europe/Northern America	12	3.5	6	3.5	2	2.5	4	4.3
Other	53	15.3	24	13.8	15	18.8	14	15.1

During the study period we performed on average 15 amniocenteses per year due to possible *T. gondii* infection during pregnancy with a decreasing tendency over time. The retrospective assessment concluded that 173 women (50.0%) were infected before pregnancy, 80 (23.1%) were possibly infected during pregnancy and 93 (26.9%) were certainly infected during pregnancy. The serological profiles indicated that 49 (14.2%) women seroconverted, 42 (12.1%) had IgG antibody increase and 255 (73.7%) women had IgM positivity and low IgG avidity/high dye test titre (Table 2).

**Table 2:** Retrospective assessed time of maternal toxoplasma infection according to maternal serologic group. Number of infected fetuses in [ ]

	Seroconversion	IgG increase	IgM positivity and low IgG avidity	n (%)
Infected before pregnancy	-	-	173	173 (50.0)
Possibly infected during pregnancy	-	-	80	80 (23.1)
Certainly infected during pregnancy	49 [11]	42 [2]	2 [2]	93 (26.9) [15]
Total *n* (%)	49 (14.2)	42 (12.1)	255 (73.7)	346 (100)

In total, 15 (4.3%) fetuses were infected during pregnancy, of which 14 had a positive toxoplasma PCR in amniotic fluid (Table 3). In the single amniotic PCR-negative pregnancy, maternal infection was suspected at gestational week (GW) 17 (Table 3). Intrauterine infection was diagnosed retrospectively based on serology and PCR results at birth and during infancy.

**Table 3:** Neonatal outcome of 15 fetuses infected with *Toxoplasma gondii*, in relation to maternal serologic group

Year of AC	Serologic group	Time of detected infection	Gestational age at AC	PCR in amniotic fluid	Antiparasitic treatment	Ultrasound findings	Gestational age at birth	Birth weight	Findings after birth
		Trimester (weeks)	Weeks		Drugs		Weeks	Gram	
1994	Sc	3 (29.1)	31.0	+	Sp, PS	–	39.0	3830	I.c. calcifications
1995	Sc	2 (19.4)	29.4	+	PS	Small BPD	30.3	1500	I.c. calcifications, hydrocephaly, chorioretinitis
1995	Sc	2 (26.6)	35.6	+	Az, PS	–	40.9	3700	Chorioretinitis
1995	Sc	2 (27.0)	29.3	+	?	?	31.4		?
1995	Sc	3 (33.9)	36.3	+	PS	–	31.4		No findings
1995	Sc	3 (36.3)	37.7	+	Az	–	38.9	3880	I.c. calcifications, parasites in placenta and CSF
1999	Sc	3 (28.0)	30.4	+	Sp, PS	I.c. calcifications, splenomegaly	41.9	3450	Chorioretinitis, leptomeningeal changes on CT
2000	Sc	3 (34.0)	35.1	+	Sp, PS	Splenomegaly, i.c. calcifications, renal pyelectasy	37.4	3268	I.c calcifications, operated because of atrial septal defect
2000	Sc	3 (35.3)	37.0	+	PS	I.c. calcifications, splenomegaly	42.1	3510	No findings
2002	Sc	2 (25.9)	28.0	+	Az, Sp, PS	–	39.9	3650	No findings
2005	Sc	3 (35.3)	37.7	+	Az, Sp, PS	I.c. calcifications, splenomegaly	38.3	3335	Jaundice, reduced vision one eye
1997	Ti	2 (14.7)	17.0	+	Sp	–	17.7	204	Termination. Parasites at autopsy; granulomas, paraventricular micronecrosis and *T.gondii* cysts in CNS. Lymphoid cells and macrocyts in lungs, necrosis and cysts in placenta
1999	Ti	1 (8.6)	13.7	+	Az	Enlarged nuchal translucency	15.4	44	Planned termination but spontaneous fetal death. Parasites at autopsy found in CNS, lungs, liver and placenta. *T.gondii* cysts in lungs and placenta
1993	IgM+/IgGav	1 (11.7)	16.4	+	Sp	–	40.6	3695	No findings
2001	IgM+/IgGav	2 (17.6)	20.4	–	Az	–	40.0		IgM, PCR of placenta and umbilical cord blood positive at birth, IgA increase after 6 months and IgG increase first year of life

*Ac* amniocentesis, *Az* azitromycin, *BPD* biparietal diametre, *CNS* central nervous systeme, *CSF* cerebro spinal fluid, *I.c.* Intra cerebral, *PCR* polymerase chain reaction, *PS* pyrimethamine-sulpha, *Sc* seroconversion, *Sp* spiramycin, *Ti* Titre/antibody increase, *IgM+/IgGav* IgM positie with low IgG avidity, *?* missing information

In the 15 pregnancies with confirmed fetal infection, 11 women had seroconverted, 2 exhibited an IgG-antibody increase and 2 showed IgM positivity and low IgG avidity. A range of clinical manifestations was observed at prenatal ultrasound or after birth in 10 of the 15 (67.0%) infected offspring (Tables 3 and 4).

**Table 4:** Ultrasound findings and outcome in 346 offspring with suspected maternal *Toxoplasma gondii* infection

	Non-infected offspring *n* = 331	Infected offspring *n* = 15
Ultrasound abnormalities	19	6
Intracerebral calcifications		3
Small biparietal diameter	3	1
Increased nuchal translucency		1
Ventriculomegaly, holoprosencephaly, cleft lip/palate	1	
Renal pyelectasy, splenomegaly, intracerebral calcifications		1
Chromosomal markers: Absent nasal bone, choroid plexus cysts, single umbilical artery	6	
Asymmetric growth, growth restriction	5	
Renal pyelectasy	2	
Oligohydramnios	1	
Abnormalities, but details not given	1	
Missing information	26	
Positive PCR		14
Miscarriage	1	1
Termination of pregnancy	2	1
Stillbirth	1	

Maternal antiparasitic treatment had been given in all cases with manifestations.

The offspring were infected in 11 of the 49 (22.4%) mothers with seroconversion compared to 2 of the 42 (4.8%) mothers with antibody increase and 2 of the 255 (0.8%) mothers with IgM positivity and low IgG avidity.

All of the infected fetuses were in the group of “certainly infected during pregnancy”.

**Fig. 1:**
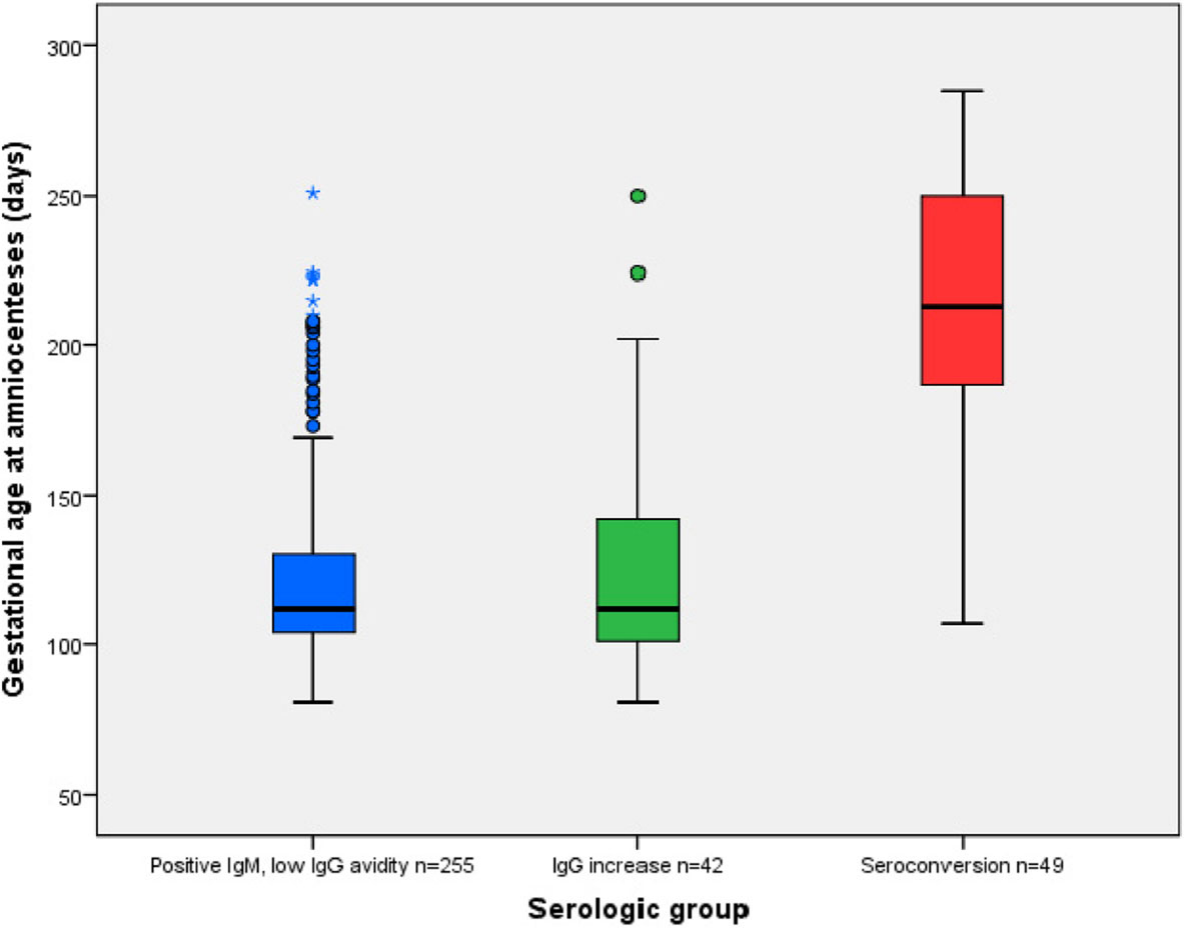
Gestational age at amniocentesis for suspected *Toxoplasma gondii* infection, according to maternal serology

The median GA at the first toxoplasma antibody test during pregnancy was 10.7 GWs (Q_1_ = 8.6, Q_3_ = 13.4), and did not differ between the three serological groups (*p* = 0.07). At the second serologic test and at amniocentesis, the GA was significantly higher for those with seroconversion (*p* = 0.001) (Fig. 1). Seroconversion was detected at a median of 27.7 GWs (Q_1_ = 22, Q_3_ = 33.9).

Amniocenteses was performed at a median of 16.7 GWs (Q_1_ = 15, Q_3_ = 22), with a median sample volume of 4 ml (Q_1_ = 3, Q_3_ = 7). All patients had a detailed fetal ultrasound examination at the time of amniocentesis, and 77.6% (242/311) received a follow-up ultrasound examination in third trimester. Ultrasound abnormalities were identified in 25 fetuses (7.2%), and 6 of these fetuses were infected. The ultrasound findings and pregnancy outcomes are presented in Tables 3 and 4.

Mean GA at birth in the uninfected fetuses was 40.0 GWs (SD 2.0) compared to 38.3 GWs (SD 3.6) in the infected fetuses, *p* = 0.003 (95% CI 0.6, 2.8).

Almost all (98.4%, 302/307) of the women were treated with antiparasitic drugs, for a median of 21 days (Q_1_ = 21, Q_3_ = 28), most commonly with azithromycin (86%) or spiramycin. In 29 women the treatment was changed to the pyrimethamine-sulphadiazine regime post-amniocentesis.

Two miscarriages occurred: one with a severely infected fetus and the other with a non-infected fetus, both 4 weeks after the procedure (Table 4). Amniocentesis (obtaining 13 ml of normal-coloured fluid) was performed at GW 13.7 in the first case; the woman had had one previous miscarriage. In the other case the amniocentesis was performed at GW 13.3, obtaining 5 ml of normal-coloured fluid in a woman with no previous miscarriages. In addition, one intrauterine fetal death was recorded at GW 28. This fetal death was detected at the scheduled amniocentesis. The infection most likely occurred prior to conception.

There were three pregnancy terminations with the following characteristics (Table 4): (i) positive PCR in the amniotic fluid, pathological ultrasound findings and infection confirmed by autopsy;

(ii) terminated pregnancy due to chromosomal aberration (and negative toxoplasma PCR); and (iii) pregnancy terminated at GW 15 upon patient request because of psychological distress due to the possibility of toxoplasma infection (negative PCR).

Serology results were recorded in 82% of the children at birth and in 42% during the first year of life.

## Discussion

We aimed to determine retrospectively whether we had performed amniocenteses in the correct patients. We observed that maternal infection occurred prior to conception in 50% of the cases. Primary maternal infection during pregnancy was identified in 26.9% of the women, and infection during pregnancy could not be ruled out in 23.1%. For most of the infected fetuses, the maternal infection was detected in late second trimester, whereas most cases of infection prior to conception were tested late in the first trimester.

One possible explanation for the high proportion of preconceptionally infected women undergoing amniocentesis is misinterpretation of the serological tests. A possible solution to avoid amniocentesis in the group with preconceptional infection is to perform an additional serological test 3–4 weeks after the second test if the test result is indeterminate. In other words, amniocentesis should only be performed in those with increasing IgG antibody or IgG avidity levels, since this indicates an ongoing immunological process [12].

The cut-off values for low IgG avidity in our study were in accordance with the manufacturer’s recommendations and similar to values used in previous studies [19, 22, 23]. However, the cut-off values may have been too high, resulting in unnecessary amniocenteses in women with latent infection. Lowering of the IgG avidity cut-off value could contribute to fewer unnecessary amniocenteses but increase the risk of underdiagnosing infected women.

Interpreting toxoplasma serology may be difficult, as illustrated by 23.1% of the women in our study retrospectively being categorised as possibly infected during pregnancy. Amniocentesis in women with latent infection is probably not always avoidable and occurs even in countries with antenatal screening programs [24]. The toxoplasma serology should be interpreted by dedicated staff, preferably a cooperation between obstetricians and microbiologists, in order to reduce unnecessary amniocenteses, antiparasitic treatment, postnatal follow-up and parental worries [25]. In addition, in at-risk groups the first toxoplasma serology should be obtained as early in pregnancy as possible.

Congenital infection was confirmed in 4.3% of the 346 offspring, which is a lower rate than found in larger studies performed in France and Austria [3, 24]. In the French study, 24.7% of the fetuses were infected with the parasite, but maternal infection was confirmed as an increase in IgG or seroconversion prior to amniocentesis. In the Austrian study based on the Austrian Toxoplasmosis register, 11.8% of the fetuses were infected. However, 45.4% of the women were considered to be infected prior to pregnancy and were excluded from the analyses. It is notable that the results of these two studies differed markedly despite the application of screening programs. If we apply similar criteria as the two studies, i.e. including only seroconversion and antibody increase, the infection rate was 14.3% (13 out of 91).

Despite only 14 having positive PCR in the amniotic fluid antenataly, 29 women received pyrimethamine-sulphadiazine, most of them exhibiting seroconversion or IgG antibody increase. Starting the treatment regime when PCR was negative, was occasionally done during the nineties due to an old protocol, but is as a rule not done any more.

A cross-sectional study performed in Norway during 1992–1994 included 60% of the pregnant population, and investigated the diagnostics and epidemiology of *T. gondii* [1, 26]. Eight of the 15 infected children in our study were identified during that period and the subsequent 3 years. No fetal infection in women undergoing antenatal amniocentesis have been detected the last 8 years despite an increase in the number of maternal samples sent to the Toxoplasma Reference Laboratory [27]. This finding could be explained by several factors, including reduced incidence, missed diagnoses due to misinterpretation of serological tests, scepticism in the population or the health-care providers towards amniocentesis, or patients at risk not being detected [1].

The prevalence of toxoplasma IgG and incidence of congenital toxoplasma infections varies greatly between countries due to variations in several factors including climate, hygiene, diet, parasite type and virulence [4, 28]. The results from studies performed outside Scandinavia can therefore not be generalised to our population. The prevalence of toxoplasma IgG among pregnant women in Norway has decreased only slightly over the past 40 years: 12.6% in 1974, 10.9% in 1994 and 9.3% in 2010 [26, 29, 30]. A Norwegian study from 1994 estimated that the incidence of maternal toxoplasma infection during pregnancy was 1,4 per 1000 pregnancies in Norway and 4,6 per 1000 in the capital Oslo [1]. Based on these findings and the relatively stable toxoplasma IgG prevalence over 40 years, we expect the incidence of maternal infection in Norway during the last 20 years to be close to that in 1994, indicating that a substantial proportion of infected children are not being detected [26]. Unlike Austria, Norway does not have a registry for new-borns infected with *T. gondii*, and the current incidence of children with diagnosed congenital infection in our country is unknown [24].

The amniotic fluid PCR was negative in 1 of the 15 infected children. In a Norwegian study performed in 1998, Jenum et al. found that the sensitivity and specificity of the toxoplasma PCR method were 59 and 94%, respectively [21]. A recently published systematic review and meta-analysis of the performance of PCR in amniotic fluid found a sensitivity of 87% and specificity of 99% when performed for up to 5 weeks after a maternal diagnosis of seroconversion or an IgG increase [31]. Another study found that the sensitivity of PCR was significantly higher if the infection occurred during 17–21 GWs compared to an earlier or later GA [32]. The infected child with a negative PCR in our study was infected before 17 GWs. The negative PCR might be explained by several factors, such as test failure, eradication of parasites by treatment before the amniocentesis or delayed placental transmission resulting in parasite transmission after the amniocentesis [31, 33]. This case highlights the need to perform testing on mother and child at birth when congenital toxoplasma infection is strongly suspected, in addition to follow-up serology of the children during the first year of life. In our study most of the infants were tested at birth, but follow-up serology during the first year of life was only performed in 42%, and decreasing during the last decade. We therefore might have missed children with congenital infection and a negative PCR in the amniotic fluid. However, in most cases maternal and neonatal serology and PCR data were obtained at birth.

The proportion of clinical findings in the infected offspring was relatively high (10 out of 15) [2]. A study from Austria reported clinical manifestations in 10.6% of the infected offspring (15 out of 141), all of which had chorioretinitis and 12 had cerebral manifestations [24]. Among the group of women infected during pregnancy in our study, the first toxoplasma serology was performed in the first trimester and the second test (detecting seroconversion or an antibody increase) was performed at a median of 8 weeks later. This treatment delay may have resulted in mother-to-child transmission or more severe manifestations in the fetuses [2]. However, the transmission rate of 14.3% (13 out of 91) in this study is similar or lower than other published rates [1, 3, 24]. A European multicentre study found that treatment did not reduce mother-to-child transmission, but the infected children had less severe manifestations [34].

There were few infected infants in our study and it is relevant to ask whether patients at risk are tested and referred. Although the prevalence of toxoplasma IgG during pregnancy has been found to be higher among immigrants in Norway [26, 35], the proportion of non-Norwegians in our study (15%) was surprisingly small given the relatively large immigrant population in the South-East Health Region (13% in 1998 and 22% in 2013 of the female population of fertile age). (https://www.ssb.no/innvbef). This might indicate that this group requests testing to a lesser extent or that health-care workers are unaware of this risk profile.

Probably only one of the two miscarriages may be related to the amniocentesis (0.3%, 1 out of 343); the other is most likely due to severe fetal infection. If the 15 pregnancies with infected fetuses are removed from the equation, the figures remain the same (0.3%, 1 out of 328). Amniocentesis was performed at 13 GWs in both cases of miscarriage. At our department we have followed the international trend away from early amniocentesis, and is now only perform after 14.9 GWs due to the higher risk of fetal loss at an earlier GA [15]. Procedure-related pregnancy loss is rare, and studies have shown that second-trimester amniocentesis is safe with no significant risk of miscarriage [15, 36–39]. Amniocenteses were performed in the present study under ultrasound guidance by trained specialists, aspirating less amniotic fluid than for a genetic amniocentesis. Operator experience is associated with the prevalence of procedure-related complications [15, 40]. The smaller amount of fluid removed in our study (4 ml versus 15 ml in genetic amniocentesis) might be one reason for the low rate of fetal loss, though the statistical power is too low to draw a definitive conclusion. To our knowledge only two studies have investigated the relationship between the amount of amniotic fluid removed and the rate of fetal loss. In both studies a trend towards a lower fetal-loss rate was found. However, in the study of Cebesoy et al. the finding was not significant and in the study of Tharmaratnam and colleagues no control group was used [41, 42]. A comparison of our results with other studies on complications after amniocentesis is challenging because we performed the procedure at a wide range of GAs.

While the present study had a retrospective design, we obtained complete data on laboratory results during pregnancy as well as information on the GA at serological testing and at amniocentesis. The material was collected over a period of 20 years, which leads to a certain degree of heterogeneity within the study population. The diagnostic techniques evolved during the study period, in particular with the introduction of IgG avidity, possibly resulting in the number of amniocenteses decreasing over time. The management of women and children did not change substantially during the follow-up period, other than the introduction of the IgG avidity test and a lower rate of toxoplasma testing during the first year of life. Our laboratory has a national reference function and all the serological analyses were performed according to international recommendations. The Department of Microbiology and Section of Fetal Medicine have worked with this issue for more than 20 years. We therefore consider our results to be relevant for other centres managing obstetric patients and performing prenatal diagnostic procedures, especially in a setting where toxoplasma screening is not part of the routine antenatal program.

## Conclusions

In our low-prevalence setting about 50% of the amniocenteses were performed on women with latent infection in which antiparasitic treatment, amniocentesis and further follow-up now seem unnecessary. To decrease the number of unnecessary amniocenteses, toxoplasma serology should be interpreted by dedicated staff. We advise that a second maternal serological test should be performed 3 weeks after a suspect test result is obtained, and a further sample 3–4 weeks thereafter in cases with inconclusive serology, before performing amniocentesis. We found that amniocentesis as a diagnostic procedure after 15 GWs is safe and useful in diagnosing congenital toxoplasma infection when infection is suspected by serology. Because of the possibility of a false-negative PCR result, the necessity of serology and PCR at birth and during first year of life needs to be emphasized.

## Abbreviations

GA: Gestational age
GW: Gestational week
IgA: Immunoglobulin A
IgG: Immunoglobulin G
IgM: Immunoglobulin M
PCR: Polymerase chain reaction
